# Effect of Smart Ambient Bright Light (SABL) in Nursing Home Residents with Dementia: A Pilot Study

**DOI:** 10.1002/alz.087626

**Published:** 2025-01-09

**Authors:** Ying‐Ling Jao, Julian Wang, Yo‐Jen K Liao, Diane Berish, Marie Boltz, Margaret Calkins

**Affiliations:** ^1^ Pennsylvania State University, University Park, PA USA; ^2^ IDEAS Institute, Cleveland Heights, OH USA

## Abstract

**Background:**

Neurobehavioral distress significantly impacts people with dementia. Lighting interventions have shown positive effects in this population, yet most did not accommodate natural daylight. This study developed an innovative, bright light system, the smart ambient bright light (SABL), that provides auto‐controlled, consistent indoor lighting and accommodates natural daylight. This study evaluated the effect of the SABL on neurobehavioral symptoms and affect in nursing home (NH) residents with dementia.

**Method:**

This crossover, cluster randomized control trial recruited residents with dementia and agitation from two NHs in Pennsylvania. The study had five phases: baseline, intervention, post‐intervention/washout, control, and post‐control/washout. The intervention and control phases were four weeks each, and the intervention sequence was randomly assigned. The SABL was installed in residents’ bedrooms and common areas. Neurobehavioral symptoms and affect were measured at baseline and biweekly. The Neuropsychic Inventory (NPI) was calculated as a total score and four subscales: psychosis hyperactivity, mood, and frontal. Repeated measures analysis of variance were conducted.

**Result:**

27 NH residents completed the study. Participants averaged 87.4 years and were primarily female (74.1%). After controlling for age, dementia stage, and gender, participants’ scores on NPI total (p = 0.007), hyperactivity (p = 0.04), mood (p = 0.03), and psychosis (p = 0.02) were statistically significantly lower during the intervention period as compared to the control period. Participants’ affect did not show a significant difference. No intervention‐related adverse effects were reported. Moreover, NH staff reported favorably that they would like the SABL to be used in their facility with an average rating of 4.1 on a 1‐5 rating scale.

**Conclusion:**

Overall, results suggest that the SABL is well‐accepted and has a positive impact on most neurobehavioral symptoms in NH residents with dementia. Future studies may replicate the study in a larger, more diverse population, examine the intervention fidelity, and establish implementation strategies in NHs.

